# What can we learn from administrative benefits data?

**DOI:** 10.23889/ijpds.v8i2.2334

**Published:** 2023-09-14

**Authors:** Juliet-Nil Uraz`, Mary-Alice Doyle, Magdalena Rossetti-Youlton

**Affiliations:** 1 Policy in Practice, London, United Kingdom; 2 London School of Economics, London, United Kingdom

We present the opportunities and limitations of administrative benefits data held by local authorities for data linkage projects. Whilst the richness of this data has been exploited by practitioners for administration, its potential remains little explored by researchers. We discuss data quality, sample selection and legal gateways for data sharing.

Drawing on our experience working with over 40 local authorities, we present the structure of three datasets: the Council Tax Reduction Scheme, the Single Housing Benefits Extract and the Universal Credit Data Share. We show what variables are usually included, under which legal gateways this data can be shared and how the cohorts represented within the data compare with the low-income population. We discuss how these datasets can be linked at the household level with a number of other data held by local authorities such as social rent and Council Tax arrears, Housing Benefit overpayments and Discretionary Housing Payments (DHPs).

Administrative benefits data provides a comprehensive snapshot of a household’s financial situation. Local authorities can proactively use and share this data with external data processors to fulfil their statutory duties if a legal gateway allows. By identifying households at risk of cash shortfalls before they reach a crisis point, councils can target support when administering local welfare schemes and preventing homelessness. By assessing eligibility for benefits, they can run data-driven uptake campaigns. This data captures a proportion of the population on national and local benefits within a local authority at several points in time. Attrition is of concern since households may leave datasets over time. Some will see their income rise and no longer qualify for benefits. Others will move out of the constituency.

Local authorities routinely process longitudinal data on households receiving means-tested benefits by administering housing benefits, council tax support, and discretionary support funds. This data provides a unique real-time insight into the socioeconomic situation of low-income households. Yet, we show that its promising potential for policy research remains largely untapped.

